# Benefit of the measurement of mesorectal extension in patients with pT3N1-2 rectal cancer without pre-operative chemoradiotherapy: Post-operative treatment strategy

**DOI:** 10.3892/etm.2012.858

**Published:** 2012-12-14

**Authors:** YOSHITO AKAGI, KAZUO SHIROUZU, SHIN FUJITA, HIDEKI UENO, YASUMASA TAKII, KOJI KOMORI, MASAAKI ITO, KENICHI SUGIHARA

**Affiliations:** 1 Department of Surgery, Kurume University School of Medicine, Fukuoka;; 2 Colorectal Surgery Division, Department of Surgery, National Cancer Center Hospital, Tokyo;; 3 Department of Surgery, National Defense Medical College, Saitama;; 4 Division of Surgery, Niigata Cancer Center Hospital, Niigata;; 5 Department of Gastroenterological Surgery, Aichi Cancer Center Hospital, Aichi;; 6 Colorectal and Pelvic Surgery Division, Department of Surgical Oncology, National Cancer Center Hospital East, Chiba;; 7 Department of Surgical Oncology, Graduate School, Tokyo Medical and Dental University, Tokyo, Japan

**Keywords:** rectal cancer, mesorectal extension, TNM staging system, prognosis, adjuvant treatment

## Abstract

A treatment strategy based on the distance of mesorectal extension (DME) for pT3N1-2 rectal cancer patients without pre-operative chemoradiotherapy has not yet been defined. The present study aimed to describe the benefit of the measurement of mesorectal extension in stratifying treatment for pT3N1-2 rectal cancer patients. Data from 512 patients with pT3N1-2 rectal cancer undergoing curative surgery at 28 institutes were analyzed in this study. DME was measured histologically, and the optimal prognostic cut-off point of the DME was determined using Cox regression analyses. Survival was calculated using the Kaplan-Meier method. The patients were subdivided into two groups based on the optimal prognostic cut-off point: DME ≤4 mm and DME >4 mm. The DME was found to be a powerful independent risk factor for predicting distant and local recurrences. The recurrence-free 5-year survival rates of patients with DME >4 mm were significantly poorer for Stages IIIB (53.3%; p=0.0015; HR, 1.76; 95% CI, 1.233–2.501) and IIIC (32.9%; p=0.0095; HR, 1.64; 95% CI, 1.119–2.407) than for patients with DME ≤4 mm (69.7 and 50.4%, respectively). The cancer-specific survival rates of patients with DME >4 mm were also significantly worse than those with DME ≤4 mm. A value of 4 mm provides the best cut-off point for subdividing the mesorectal extension to predict oncologic outcomes. Measurement of mesorectal extension appears to be of benefit in stratifying patients for post-operative adjuvant treatments.

## Introduction

It is currently unknown whether the distance of mesorectal extension (DME) is applicable as a parameter for adjuvant treatment and is associated with the prognosis of rectal cancer. In 1990, the clinical importance of subdividing the mesorectal extension at a cut-off point of 4 mm was advocated (1). In 1993, the International Union Against Cancer (UICC) proposed optional subdivisions for pT3 and pT4 tumors (2). Thereafter, several studies have shown the prognostic heterogeneity of pT3 rectal cancers (3–12). However, appropriate treatment strategy for T3/pT3 rectal cancer based on the DME remains unclear. In European countries, the standard strategy for T3 rectal cancer is preoperative chemoradiotherapy (CRT) (13,14). However, not all T3 rectal cancers are necessarily suitable for CRT. Moreover, it is considerably difficult to evaluate not only DME but also conventional prognostic factors such as lymphatic, venous and perineural invasion in pathological specimens following preoperative CRT. When preoperative CRT is not administered to certain patients with T3 rectal cancers, it appears to be vital to accurately assess the DME and to evaluate the prognosis following surgery. In the current study, we analyzed a large collection of data obtained from a multi-institutional study promoted by the Japanese Society for Cancer of the Colon and Rectum (JSCCR). This study confirms the benefit of the measurement of mesorectal extension and selection of patients for postoperative adjuvant treatment strategy in pT3N1-2 rectal cancers based on the TNM classification (6th edition) (15,16).

## Patients and methods

### Patients

Approval from the ethics committees of both the JSCCR and the local Institutional Review Board were obtained in order to review medical records and to permit follow-up patient contact. However, informed consent could not be obtained from all patients, since this study was retrospective and some patients may be deceased. Data were obtained from 1009 patients with pT3 rectal cancer from 28 institutes that are members of the Study Group of the JSCCR on Extramural Mesorectal Extension of Rectal Cancer. All patients had a primary rectal adenocarcinoma located in the middle or lower rectum. Patients with rectosigmoid colon cancer were not included in this study. Histologically defined curative surgery was performed between 1995 and 1999. Patients undergoing non-curative surgery (R2 operation) were excluded from this study. Patients were staged according to the pathological TNM classification (6th edition) (15,16). The present study focused on postoperative treatment strategy in patients with Stage III (pT3N1-2) disease. After staging, 512 patients with Stage III disease remained enrolled in this study, including 321 with Stage IIIB and 191 with Stage IIIC diseases. Clinicopathological information was available and eligible for analysis. Neither radiotherapy nor neoadjuvant chemotherapy was performed prior to surgery in these enrolled patients. All 512 patients underwent total mesorectal excision. According to the postoperative adjuvant treatment protocol of each institute, peroral 5-fluouracil (5-Fu)-based chemotherapy, such as 5′-DFUR (doxifluridine), HCFU (1-hexylcarbamoyl-5-fluorouracil), or UFT (tegafur-uracil) were most frequently administrated. Clinicopathological data and follow-up system were based on the Japanese rules defined by the JSCCR (17). The follow-up system consisted of the measurement of serum tumor markers, chest X-ray and abdominal ultrasound examination every three months for the first three years, and then every six months for the next two years. When the development of recurrence was suspected by serum tumor markers, digital examination and/or ultrasonography, the final diagnosis was carried out using rectoscopy, computed tomography (CT) and/or magnetic resonance imaging (MRI) and other diagnostic tools. Local recurrence was defined as the presence of a radiologically confirmed or histologically proven tumor non-hematogenously occurring in the pelvis within the field of the initial surgery. Distant metastasis included hematogenous metastases to the liver, lung, bone, brain, kidney or other organs. The other-organ recurrences were defined as recurrence other than local recurrence or distant metastasis, i.e., peritoneal dissemination, intra-abdominal, para-aortic, subclavicular, mediastinal and inguinal lymph node metastases. The outcomes of all patients were investigated in detail. From January 1995, eligible surviving patients were followed for a median of 86 months (range, 1–166).

### Measurement of mesorectal extension

All surgically resected specimens were opened along the anti-tumor or anti-mesenteric side. Specimens were fixed in 20% formalin for at least 48 h after being pinned to a wood or cork board. One or more longitudinal sections of the tumor were sliced at the point of maximum extramural invasion and were embedded in paraffin after being divided into blocks of suitable size. These tissue blocks were then routinely processed for hematoxylin and eosin and Elastica Von Gieson staining. Tumor category pT3 sections were subdivided based on the histological measurement of the maximum depth of invasion beyond the outer border of the muscular layer (in mm). Without any knowledge of clinical information, histological measurement was performed according to our previous methods (18). When the outer border of the muscular layer was completely identifiable (sometimes identifiable as fragments of muscle), the distance from the outer border of the muscular layer to the deepest part of the invasion was measured. When the outer border of the muscular layer was not entirely identifiable, due to destruction by the invasion or excessive inflammatory reaction, an estimate of the outer border was obtained by drawing a straight solid line between both break points in the muscular layer.

### Statistical analysis

Statistical analysis was performed using StatView 5.0 and JMP 8.0 (SAS Institute, Inc., Cary, NC, USA) software for Windows. All clinicopathological independent variables (13 items) were coded for analysis. Overall recurrence, distant metastasis, local recurrence and survival were coded as dependent variables. Cox regression analyses were used to determine the optimal cut-off point of the mesorectal extension for postoperative recurrence. The Cox regression analysis was also used to estimate the independent risk factors for either distant metastasis or local recurrence. The Kaplan-Meier method and the log-rank test were used for calculating survival rates. Statistical significance was determined at p<0.05 and the confidence interval (CI) was determined at 95%.

## Results

### Distance of ME

The DME in these 512 cases (pT3N1-2 tumor) was measured histologically. The mean DME was 5.4±4.4 mm, and the median DME was 4.3 mm (range, 0.1–28.4).

### Postoperative recurrence pattern

Postoperative overall recurrence occurred in 247 (48.2%) of the 521 patients. A total of 55 patients (10.7%) had local recurrence only, and 124 (24.2%) had distant metastasis only. Furthermore, 30 patients (5.9%) had both local and distant recurrences. The remaining 38 patients exhibited other recurrences, that is, peritoneal dissemination, intra-abdominal, para-aortic, subclavicular, mediastinal and inguinal lymph node metastases.

### Cut-off point for subdividing mesorectal extension

The multivariate Cox regression analyses for recurrence-free survival are shown in Table I. A cut-off value of 4 mm showed the highest Chi-square (17.463), lowest p-value (p=0.00003), and high hazard ratio (HR, 1.72). The L/U ratio (lower/upper limits of CI) showed higher reliability (0.5950) among other cut-off points. A cut-off value of 4 mm was found to be the best predictor of recurrence-free survival. Overall, the best cut-off point was determined to be 4 mm, therefore, the patients were divided into two groups according to mesorectal extension: ≤4 mm and >4 mm.

### Independent risk factors for distant metastasis and local recurrence

Distant and/or local recurrence-related independent variables used for analyses are listed in Table II. Multivariate Cox regression analysis showed that the DME was a powerful independent risk factor for distant metastasis (HR, 1.82; 95% CI, 1.300–2.538; p= 0.0005) and for local recurrence (HR, 1.74; 95% CI, 1.107–2.744; p=0.0164).

### Distant metastasis and local recurrence based on the cut-off value

Stage-specific distant metastasis and local recurrence occurred in 86 (26.8%) and 40 patients (12.5%), respectively, at Stage IIIB, and 68 (35.6%) and 45 patients (23.6%), respectively, at Stage IIIC (Table III). Taking into account the cut-off value of 4 mm, the rates of distant metastasis at IIIB and IIIC were significantly higher (32.1 and 41.9%, respectively) in patients with DME >4 mm compared to patients with DME ≤4 mm (21.4 and 27.9%, respectively). Local recurrence showed a trend toward a higher rate at the cut-off value at any Stage.

### Recurrence-free and cancer-specific survival rates

**Figure 1.**
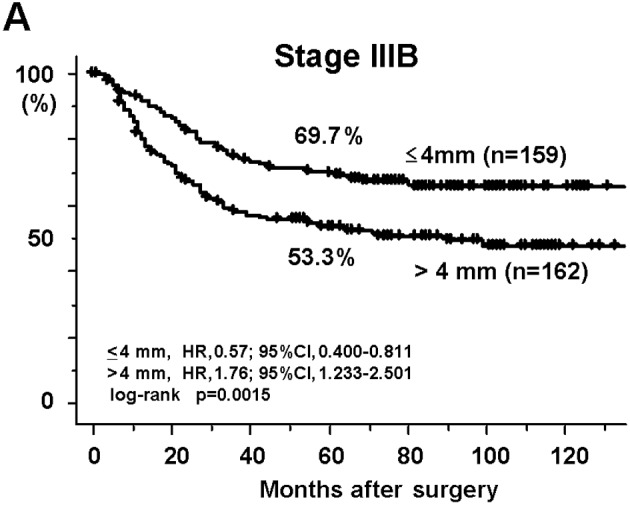

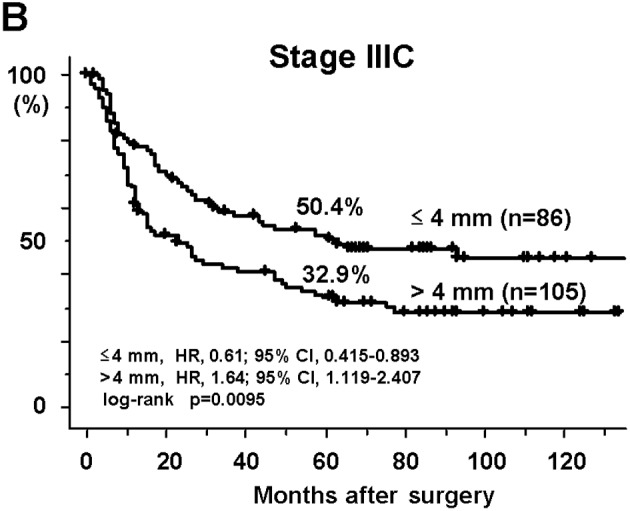
Recurrence-free 5-year survival. The recurrence-free 5-year survival rates of patients with DME >4 mm were significantly poorer at Stages (A) IIIB (53.3%; p=0.0015) and (B) IIIC (32.9%; p=0.0095) than those of patients with DME ≤4 mm. DME, distance of mesorectal extension.

**Figure 2.**
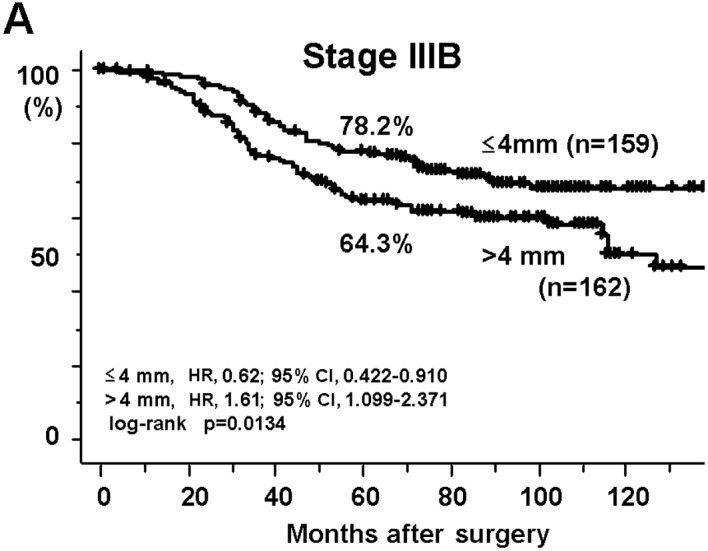

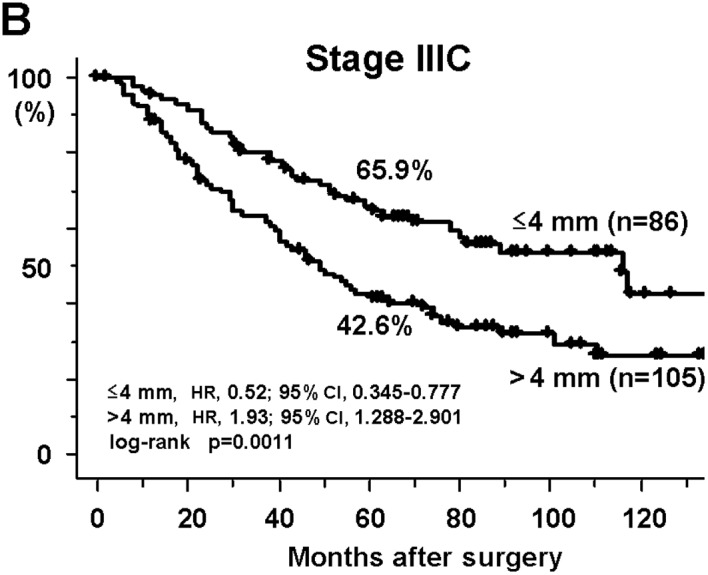
Cancer-specific 5-year survival. The cancer-specific 5-year survival rates of patients with DME >4 mm was significantly worse at Stages (A) IIIB (64.3%; p=0.0134) and (B) IIIC (42.6%; p=0.0011) than those of patients with DME ≤4 mm. DME, distance of mesorectal extension.

The recurrence-free 5-year survival rates of the DME >4 mm group were significantly worse [53.3% at Stage IIIB (HR, 1.76; 95% CI, 1.233–2.501; p=0.0015) and 32.9% at Stage IIIC (HR, 1.64; 95% CI, 1.119–2.407; p=0.0095)] than those of the patients with a DME ≤4 mm (69.7 and 50.4%, respectively; Fig. 1A and B). The cancer-specific 5-year survival rates of the DME >4 mm group were also significantly worse at Stage IIIB (64.3%; HR, 1.61; 95% CI, 1.099–2.371; p= 0.0134) and at Stage IIIC (42.6%; HR, 1.93; 95% CI, 1.288–2.901; p=0.0011) than those of patients with a DME ≤4 mm (78.2 and 65.9%, respectively; Fig. 2A and B).

## Discussion

In the early 1990s, the clinical importance of subdividing the mesorectal extension for pT3 and pT4 tumors was advocated (1,2). Thereafter, the importance was reported by several authors, who showed the prognostic heterogeneity of pT3 rectal cancers (1,3,5–11). At a cut-off point of 4 mm, the DME >4 mm was confirmed as an independent adverse prognostic factor for survival using multivariate analysis (1,7,8). Other authors found prognostic heterogeneity of N1-2 tumors between pT3a (≤5 mm) and pT3b (>5 mm) groups (4) and reported prognostic heterogeneity of pT3N1-2 tumors at the cut-off point of 6 mm from two different patient databases (6). Thus, the majority of studies found prognostic heterogeneity of mesorectal extension in pT3 rectal cancers at various cut-off points. However, the clinical significance and statistical appropriateness of these cut-off points remain controversial, partly because these studies had small sample sizes with underpowered statistical analyses and included cohorts from only a single institution. Based on the statistical analyses in the present study, the appropriate prognostic cut-off point was theoretically set at a value of 4 mm, and the patients were divided into two groups based on mesorectal extension: DME ≤4 mm and DME >4 mm. A recent multi-institutional study by our group demonstrated that a cut-off point of 4 mm independently delineated adverse prognosis among pT3N0 rectal cancers based on TNM classification (6th edition) (18). However, the appropriate postoperative treatment strategy for pT3N1-2 rectal cancers based on the DME remains unclear.

Dutch and Swedish trials have reported that preoperative CRT has decreased local recurrence rate to 15% in Stage III rectal cancers (13,14), which is similar to our data without using preoperative CRT. However, there have been only a few reports on the correlation of DME and local recurrence. Merkel *et al*(4) reported that the local recurrence rate was significantly higher in pT3b tumors with DME >5 mm (N1–2; 34.0%) compared with pT3a tumors ≤5 mm (N1–2; 17.1%). Another study did not find any correlation between local recurrence and DME (6). Our data showed no significant difference with regard to stage-specific local recurrence at the cut-off point in any stage due to the small number of patients. Overall, our study indicates that local recurrence occurs at a high rate in Stage III patients with a DME of >4 mm (p=0.0132, Table III).

Multivariate Cox regression analysis showed that the DME was an important parameter to predict distant and local recurrences, and was more effective than conventional prognostic parameters such as lymphatic invasion, venous invasion, circumferential resection margin, and total number of retrieved lymph nodes (Table II). As the DME becomes deeper, it is considered that undetectable lymphovascular invasions or micro-tumor deposits increase in the mesorectal adipose tissues. These isolated tumor cells may cause local recurrence and/or distant metastases. In European countries, preoperative CRT is the standard strategy for selected patients with T3 rectal cancer to eradicate those isolated tumor cells and to control local recurrence. However, it is considerably difficult to evaluate not only DME but also those pathological parameters following preoperative CRT. The current study also determined that the DME was a useful predictor to estimate survival rates (Figs. 1 and 2), which was similar to results reported by other authors (4,6). When preoperative CRT is not applied for some patients with pT3 rectal cancer, it appears to be vital to accurately assess the DME and evaluate the prognosis following surgery (3). In addition, the present study supported the reproducibility of a cut-off point of 4 mm even in pT3N1–2 disease as in pT3N0 disease (TNM 6th edition) (18).

Diagnostic techniques using MRI enable accurate measurement of the mesorectal extension that strongly correlates with the pathological measurement (19,20). If the cut-off value can be applied to the preoperative MRI-based diagnosis, then this would be more efficient for stratifying the appropriate patients for preoperative CRT. In the present series between 1995 and 1999, postoperative adjuvant chemotherapy was administered orally under the criteria for each institute. More intensive adjuvant treatments are required for patients with a DME of >4 mm to eradicate isolated tumor cells, prevent postoperative recurrence and improve survival.

In conclusion, a value of 4 mm provides the best cut-off point for subdividing the mesorectal extension to predict oncologic outcomes. The current study suggests that DME is a highly beneficial parameter with which to stratify patients for postoperative adjuvant treatments. However, further prospective studies are required to assure the reproducibility and validity of this cut-off point.

## Figures and Tables

**Table I.** Cut-off points of distance of mesorectal extension for recurrence-free survival using multivariate Cox regression analysis.

DME (mm)	No. of patients	RF survival at 5 years (%)	Chi-square	HR (95% CI, L-U)	L/U ratio	p-value
>1 vs. ≤1	445 vs. 67	52 vs. 64	4.174	1.53 (1.012–2.317)	0.4368	0.0411
>2 vs. ≤2	391 vs. 121	51 vs. 65	10.366	1.70 (1.224–2.370)	0.5165	0.0013
>3 vs. ≤3	330 vs. 182	48 vs. 65	14.423	1.71 (1.290–2.270)	0.5683	0.0001
>4 vs. ≤4	267 vs. 245	46 vs. 63	17.463	1.72 (1.328–2.232)	0.5950	0.00003
>5 vs. ≤5	204 vs. 308	44 vs. 60	16.331	1.67 (1.297–2.155)	0.6019	0.00005
>6 vs. ≤6	167 vs. 345	46 vs. 58	11.059	155 (1.191–2.006)	0.5937	0.0009
>7 vs. ≤7	135 vs. 377	43 vs. 58	13.061	1.63 (1.246–2.140)	0.5822	0.0003
>8 vs. ≤8	98 vs. 414	39 vs. 58	16.071	1.80 (1.341–2.407)	0.5572	0.00006
>9 vs. ≤9	79 vs. 433	39 vs. 57	12.495	1.74 (1.273–2.386)	0.5335	0.0004
>10 vs. ≤10	59 vs. 453	39 vs. 56	11.980	1.82 (1.287–2.575)	0.4998	0.0005

DME, distance of mesorectal extension; RF, recurrence-free; HR, hazard ratio; CI, confidence interval; L, lower limit; U, upper limit.

**Table II.** Independent risk factors for distant metastasis and local recurrence using multivariate Cox regression analysis.

Variable	Distant metastasis			Local recurrence		
	Rate of DM (%)	HR (95% CI)	p-value	Rate of LR	HR (95% CI)	p-value
Gender	28 vs. 31	n.a.		15 vs. 16	n.a.	
Male vs. female						
Size of tumor	26 vs. 31	n.a.		15 vs. 16	n.a.	
>5 vs. ≤5 cm						
Location of tumor	31 vs. 24	1.28 (0.845–1.947)	0.2425	11 vs. 18	1.44 (0.784–2.629)	0.2411
Lower vs. middle						
Gross type	27 vs. 29	n.a.		20 vs. 15	n.a.	
Infiltrative vs. expansive						
Histology	30 vs. 27	n.a.		15 vs. 16	n.a.	
Others vs. well						
Lymphatic invasion	30 vs. 28	n.a.		17 vs. 14	n.a.	
ly2–3 vs. ly0–1						
Venous invasion	29 vs. 29	n.a.		14 vs. 17	n.a.	
v2–3 vs. v0–1						
DME	34 vs. 24	1.82 (1.300–2.538)	0.0005	18 vs. 13	1.74 (1.107–2.744)	0.0164
>4 vs. ≤4 mm						
CRM	28 vs. 29	n.a.		14 vs. 16	n.a.	
≤1 vs. >1 mm						
Number of retrieved LN	25 vs. 29	n.a.		14 vs. 15	n.a.	
<12 vs. ≥12						
Operative methods	34 vs. 25	1.50 (1.025–2.197)	0.0370	11 vs. 20	1.97 (1.160–3.339)	0.0121
APR vs. SSO						
Autonomic nerve-saving	29 vs. 26	n.a.		16 vs. 13	n.a.	
Yes vs. no						
Chemotherapy	27 vs. 31	n.a.		17 vs. 14	n.a.	
Yes vs. no						

DM, distant metastasis; LR, local recurrence; HR, hazard ratio; CI, confidence interval; n.a., variables not selected for multivariate analyses as they were not significant in univariate analysis. Well, well-differentiated adenocarcinoma; others, moderately differentiated, poorly differentiated, and mucinous adenocarcinoma; ly0–1, v0–1, negative-to-minimal invasion; ly2–3, v2–3, moderate-to-severe invasion; DME, distance of mesorectal extension; CRM, circumferential resection margin; LN, lymph node; APR, abdominoperineal resection; SSO, sphincter-saving operation.

**Table III.** Distant metastasis and local recurrence at the cut-off value of 4 mm using Cox regression analysis.

	Distant metastasis			Local recurrence		
TNM Stage (6th edition)	No. of DM patients (%)	HR (95% CI)	p-value	No. of LR patients (%)	HR (95% CI)	p-value
Stage IIIB (n=321)	86 (26.8)			40 (12.5)		
≤4 mm (n=159)	34 (21.4)	1		16 (10.1)	1	
>4 mm (n=162)	52 (32.1)	1.79 (1.154–2.773)	0.0094	24 (14.8)	1.66 (0.878–3.151)	0.1186
Stage IIIC (n=191)	68 (35.6)			45 (23.6)		
≤4 mm (n=86)	24 (27.9)	1		16 (18.6)	1	
>4 mm (n=105)	44 (41.9)	1.82 (1.106–3.008)	0.0186	29 (27.6)	1.79 (0.964–3.331)	0.0652
Overall (n=512)	154 (30.0)			85 (16.6)		
≤4 mm (n=245)	58 (23.7)	1		32 (13.1)	1	
>4 mm (n=267)	96 (36.0)	1.83 (1.314–2.541)	0.0003	53 (19.9)	1.75 (1.125–2.736)	0.0132

DM, distant metastasis; LR, local recurrence; HR, hazard ratio; CI, confidence interval.
